# Bias-Repeatability Analysis of Vacuum-Packaged 3-Axis MEMS Gyroscope Using Oven-Controlled System

**DOI:** 10.3390/s23010256

**Published:** 2022-12-26

**Authors:** Hussamud Din, Faisal Iqbal, Jiwon Park, Byeungleul Lee

**Affiliations:** School of Mechatronics Engineering, Korea University of Technology and Education, Cheonan 31253, Republic of Korea

**Keywords:** bias stability, gyroscope, inertial sensors, MEMS, micro-oven, temperature compensation

## Abstract

The performance of microelectromechanical system (MEMS) inertial measurement units (IMUs) is susceptible to many environmental factors. Among different factors, temperature is one of the most challenging issues. This report reveals the bias stability analysis of an ovenized MEMS gyroscope. A micro-heater and a control system exploiting PID/PWM were used to compensate for the bias stability variations of a commercial MEMS IMU from BOSCH “BMI 088”. A micro-heater made of gold (Au) thin film is integrated with the commercial MEMS IMU chip. A custom-designed micro-machined glass platform thermally isolates the MEMS IMU from the ambient environment and is vacuum sealed in the leadless chip carrier (LCC) package. The BMI 088 built-in temperature sensor is used for temperature sensing of the device and the locally integrated heater. The experimental results reveal that the bias repeatability of the devices has been improved significantly to achieve the target specifications, making the commercial devices suitable for navigation. Furthermore, the effect of vacuum-packaged and non-vacuum-packaged devices was compared. It was found that the bias repeatability of vacuum-packaged devices was improved by more than 60%.

## 1. Introduction

In recent decades, microelectromechanical (MEMS) devices, precisely the inertial measurement units (IMUs), have gained tremendous importance for their wide range of applications. The global market survey shows a growing demand for inertial sensors due to their improved performance, replacing their macro counterparts in many promising applications such as consumer electronics, robotics, avionics, electronic stability control (ESC), global navigation satellite system (GNSS), and image stabilization [1,2,3,4,5,6,7]. Specifically, MEMS gyroscopes have been progressively famous for their ultra-high measurement sensitivity, high reliability, low power consumption, and low cost [2,8,9,10].

The performance and accuracy of MEMS IMUs are affected by several parameters, such as temperature, ambient vibrations, and acoustic noise [11,12,13,14]. However, regulating their temperature is complex and challenging [12,15,16]. Temperature-induced errors occur due to changes in the material properties of the selected material and electronic parts. In MEMS sensors, temperature-induced errors result in several other errors, such as spring softening, resonant frequency error, cross-axis sensitivity, and bias instability [17,18,19,20]. Among all the high-performance MEMS gyroscope specifications, bias stability is the first thing system designers must ensure to deliver performance [21]. The navigation grade, vacuum-packaged MEMS gyroscopes’ bias-repeatability improvement is significant.

Different compensation techniques for temperature effects have been used to improve the bias stability of MEMS gyroscopes. Such techniques include mechanical compensation, material variation, and electrostatic forces. However, it was noted that compensation is necessary for bias stability improvement and a controlled sensor temperature [22,23,24]. For temperature control, ovenization is an effective technique that has been implemented by attaching a local heater to the MEMS devices [25,26,27,28].

This study presents a bias-repeatability and locally integrated heater performance analysis of an ovenized vacuum-packaged commercial MEMS gyroscope from Bosch “BMI 088” [29]. A PID/PWM-based conventional heater driving circuit [20] is integrated with the MEMS device. This device was vacuum packaged to isolate it from its surroundings and reduce heat dissipation. The locally integrated heater keeps the device’s temperature stable, i.e., above the highest environmental temperature. A previously developed LabVIEW-based automatic test system was utilized for data gathering and analysis. An NI device, NI 8452 [30], was utilized for sensor interfacing through I2C.

This article is arranged as follows: Section 2 presents the ovenization and vacuum packaging of the commercial MEMS IMU “BMI 088”. Section 3 presents a detailed description of the experiment’s design. Section 4 discusses the experimental results of the “BMI 088”. Finally, the paper is concluded in Section 5.

## 2. Ovenization and Vacuum Packaging

**Figure 1:**
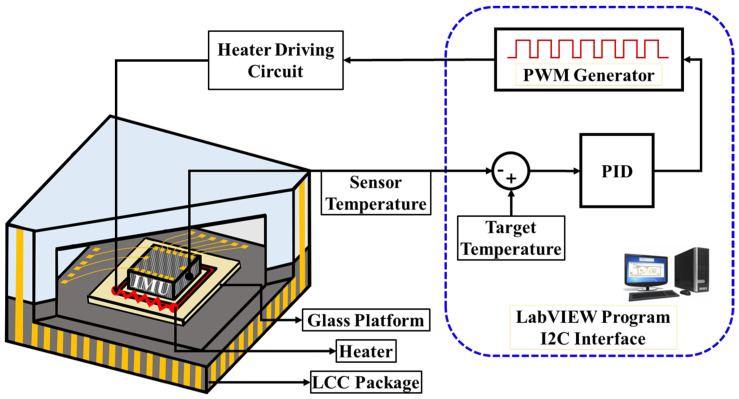
Block Diagram of the Oven-Controlled System.

Ovenization and vacuum packaging aim to provide a constant temperature to the MEMS device for performance improvement. Figure 1 shows the block diagram of the oven-controlled system for a 3-axis MEMS gyroscope. The proposed oven-controlled system consists of a heater driving circuit with a PID/PWM circuit. The PWM scheme is advantageous over conventional schemes due to its low power consumption, high power efficiency, small size, duty cycle, and digital scheme operation. The duty cycle of the fixed square wave signal controls the heater operating the current by switching the power transistors ON and OFF instead of employing a continuous current supply. The built-in temperature sensor of the MEMS IMU “BMI 088” has been used for temperature measurement. The LabVIEW program compares the IMU and target temperatures, while the PID/PWM program keeps the heater temperature constant.

**Figure 2:**
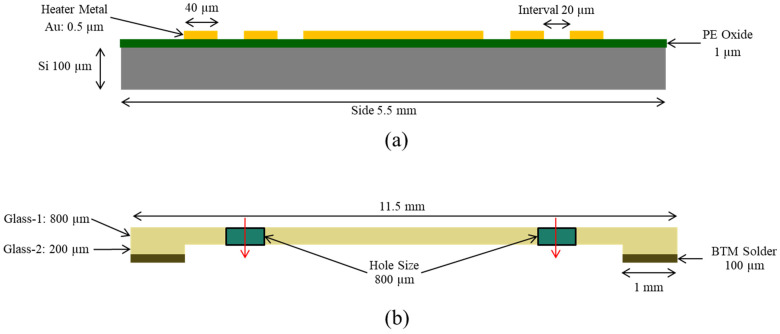
Heater Design with Glass Isolation Platform. (**a**) Heater design structure. (**b**) Glass Platform structure.

**Figure 3:**
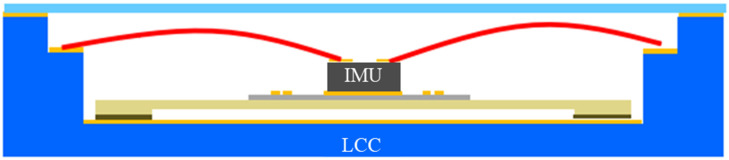
Micro-Oven and IMU Integration in LCC Package.

A thin film of gold (Au) heater is designed under the IMU chip area, and the “BMI 088” IMU is mounted on it and assembled in a leadless chip carrier (LCC) package. A custom-designed micro-machined glass platform and glass suspensions were used for isolation. The glass isolation platform provides a smooth surface for the sensor and connecting lines, having excellent thermal and mechanical properties, as shown in Figure 2 and Figure 3, respectively.

**Figure 4:**
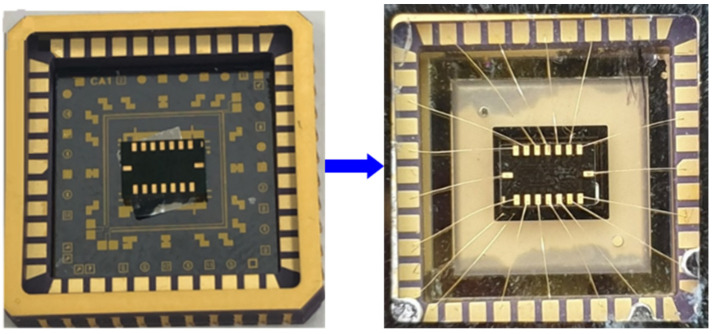
Vacuum-Packed Ovenized MEMS IMU.

The IMU has been connected through wire bonding to the metal pads, and the device has been sealed in a vacuum package, as shown in Figure 4. The vacuum level inside the package is kept at around 100 mTorr to achieve a high thermal resistance for heat loss compensation [31].

## 3. Design of Experiment

**Figure 5:**
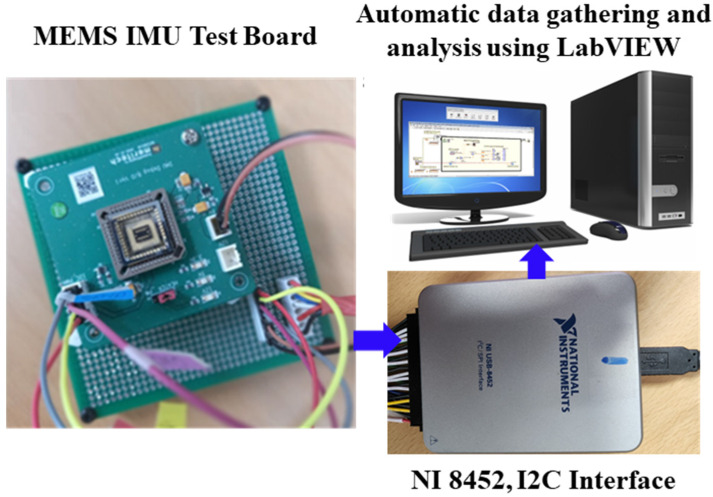
Experimental Setup.

The commercial MEMS IMU “BMI 088” was interfaced using the “NI 8452” device through the I2C protocol using LabVIEW [30,32]. The experimental setup is shown in Figure 5.

**Figure 6:**
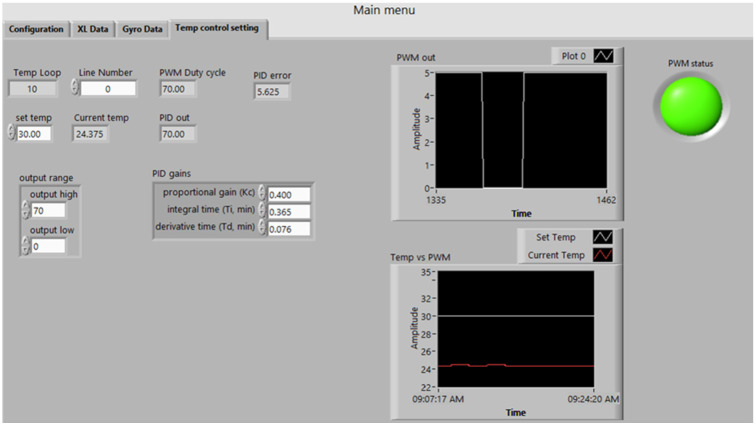
LabVIEW-Based Automatic Test System.

**Figure 7:**
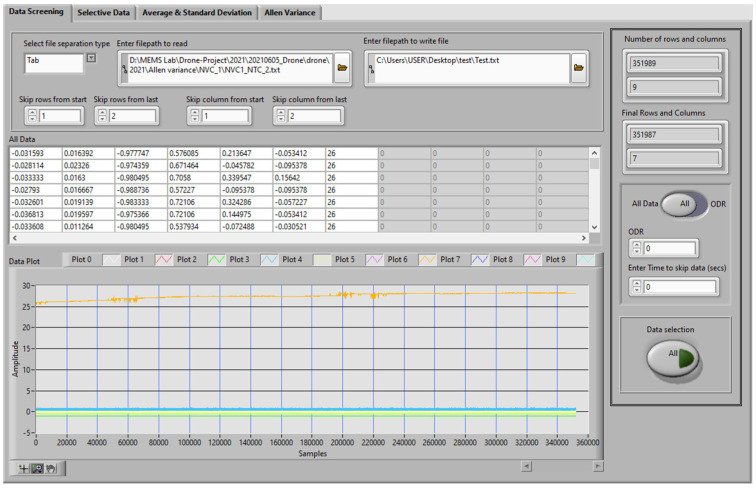
LabVIEW-Based Data Analysis Program.

In this work, a previously developed program based on the state machine and data analysis was utilized for automatic data gathering of bias-repeatability tests and data analysis programs to reduce human effort and test time. Figure 6 shows the LabVIEW program dashboard for the automatic test system, while Figure 7 shows the dashboard for the data analysis program using LabVIEW.

Initially, the heater performance and PWM duty cycle were analyzed. After that, the bias-repeatability test was conducted for three non-vacuum and three vacuum-packed ovenized devices. The resulting data were analyzed using the LabVIEW-based data analysis program.

### 3.1. Heater Performance and PWM Duty Cycle Analysis

The built-in temperature sensor of the “BMI 088” was utilized for the temperature measurement of the locally integrated heater. The PWM algorithm with a fixed square wave signal was used [33] for the locally integrated heater, and the digital I/O (DIO) lines of the “NI 8452” device were used to control the current supply [30,32]. According to the selected duty cycle and target temperature, the DIO line is turned ON and OFF by the PWM signal for heater switching and constant temperature. The relationship between the duty cycle and the maximum achievable temperature was computed according to the experiment conditions listed in Table 1.

The duty cycle varied from 0% to 80% at a DC voltage of 7 V. The duty cycle test was performed, and the data were gathered for 15 min with a delay interval of 5 min as a cool-down time. The start temperature varied every time because of the variations in environmental temperature and changes in the duty cycle.

**Figure 8:**
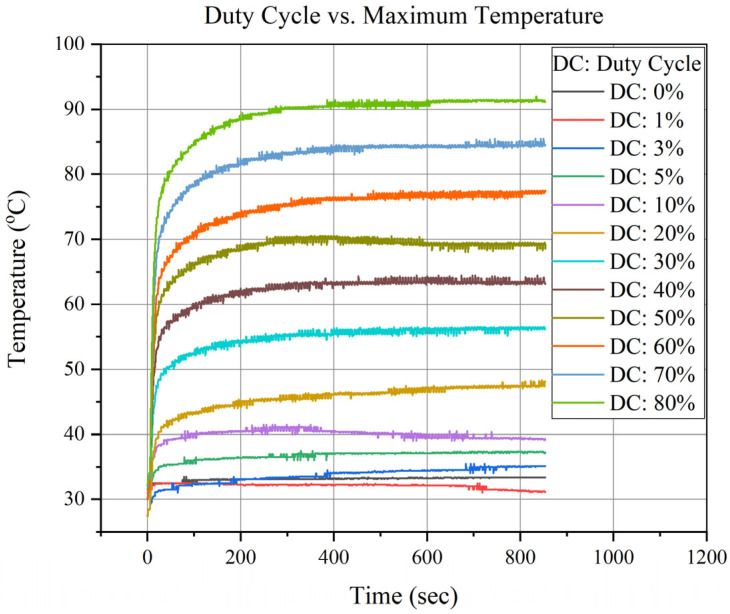
Duty Cycle vs. Maximum Temperature Analysis.

The time-domain data of the sensor temperature and duty cycle are shown in Figure 8. The variation in the actual temperature is due to the air conditioning in the room.

**Figure 9:**
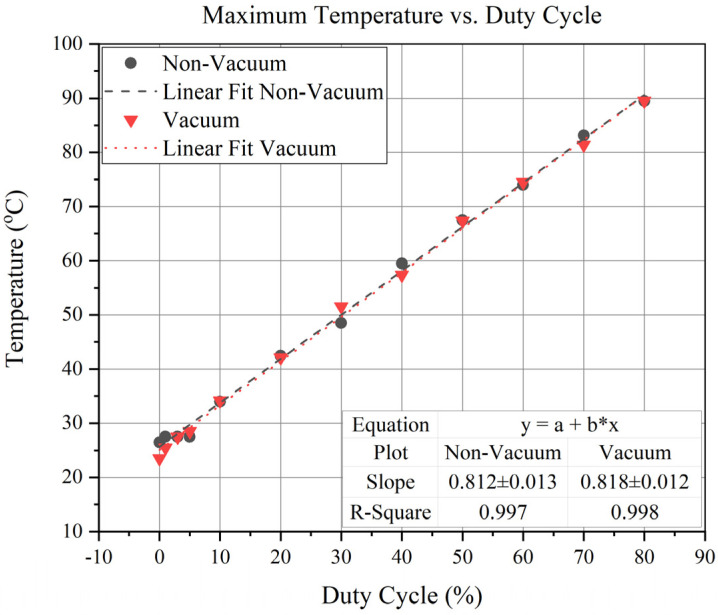
Maximum Temperature vs. Duty Cycle of Vacuum and Non-Vacuum Devices.

Furthermore, a duty cycle vs. maximum achievable temperature test was conducted for one non-vacuum and one vacuum device, respectively, as shown in Figure 9. A temperature change of 0.81 °C/duty cycle was observed. Since the glass was used as a thermal isolation platform, exhibiting excellent thermal properties, the study found that the heat loss conduction was reduced, and there is no significant change in temperature concerning the duty cycle.

### 3.2. Bias-Repeatability Analysis

**Bias-Repeatability:** Turn-on bias repeatability is measured by averaging all the data gathered by turning the sensor ON and OFF for specific time intervals. The test is conducted by turning OFF the power from the sensor. In the case of repeatability, the test is conducted several times. This kind of test is difficult for humans to handle without an automatic test system.

**Figure 10:**
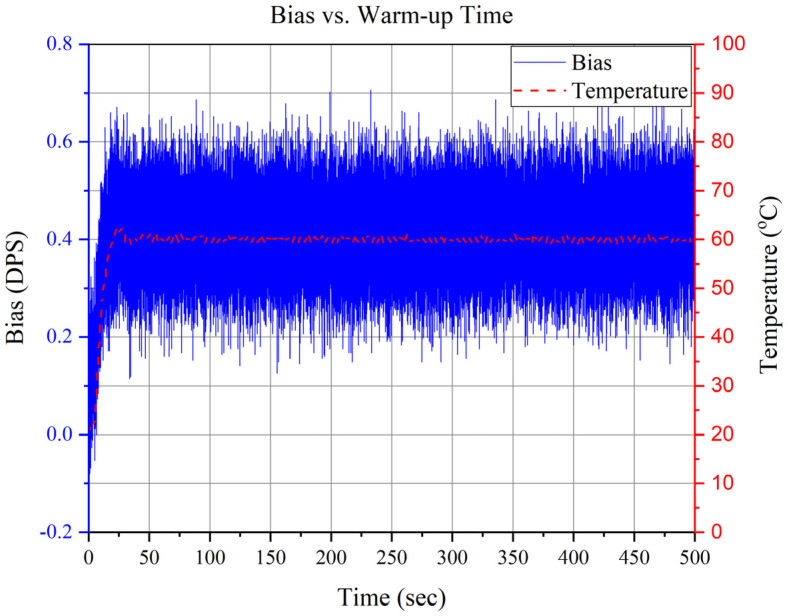
Bias vs. Warm-up Time of the Ovenized Device.

Before starting the bias-repeatability test, the bias vs. warm-up time was analyzed and was found to be 50 s, as shown in Figure 10. Turn-on bias repeatability or ON/OFF bias was measured by averaging all the data gathered for a specific interval by turning the sensor ON and OFF. The bias-repeatability test was conducted according to IEEE standards; i.e., the samples in each data set were more significant than 10,000 [34,35,36]. Each device was tested for 24 hr at room temperature, while a 60 °C controlled temperature was used in the integrated micro-oven.

During the bias-repeatability test, the sensor was turned ON for 30 min and then turned OFF for 30 min using the automatic test setup. For each device, 25 data sets were gathered for a 24 hr test. The room temperature varied from 11 °C to 20 °C in winter and from 19 °C to 28 °C during summer. Six devices were tested, three of them were vacuum packaged, and three were non-vacuum packaged. The experimental results are discussed in Section 4.

## 4. Experimental Results

**Figure 11:**
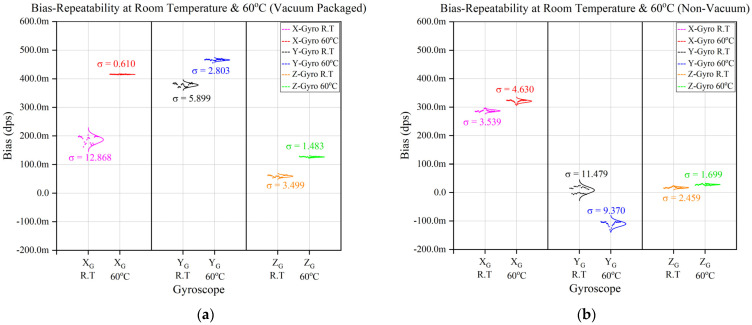
Bias-Repeatability Analysis of Vacuum- and Non-Vacuum-Packaged Devices. (**a**) Bias-Repeatability of Vacuum-Packaged Device. (**b**) Bias-Repeatability of Non-Vacuum-Packaged Device.

The 24 hr bias-repeatability test results for two devices (i.e., one vacuum packaged and one non-vacuum packaged) at room temperature, as well as at a 60°C controlled temperature, are shown in Figure 11a,b, respectively. The target of this research project was to keep the bias repeatability lower than 5.5 mdps or 20°/hr.

The experimental results for a single device, as depicted in Figure 11, show that bias repeatability was improved at a controlled temperature compared with the room temperature. However, even though a few non-vacuum devices were improved with temperature control, the results are inconsistent with vacuum-packaged devices.

**Figure 12:**
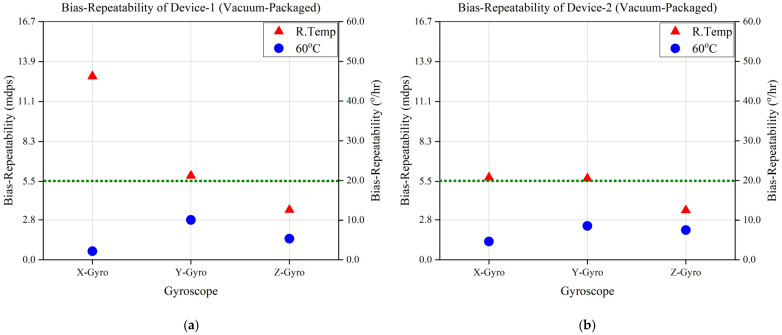

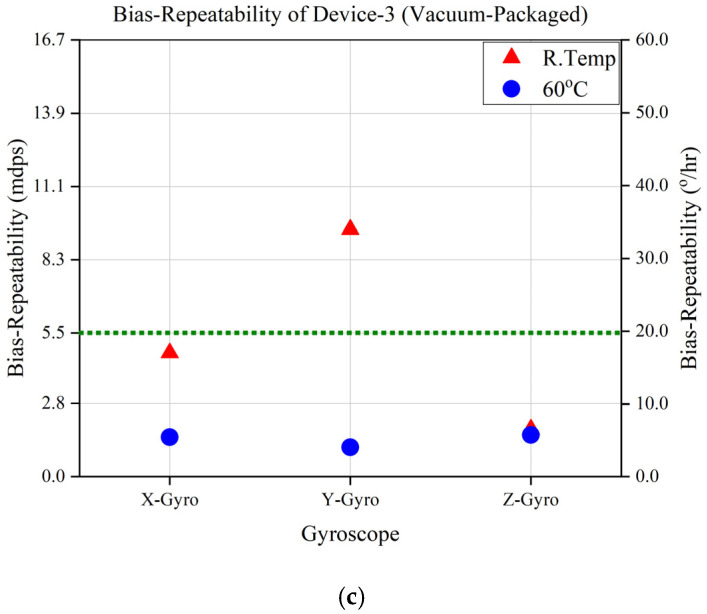
Bias-Repeatability Analysis of Vacuum-Packaged Devices at Room Temperature and 60 °C. (**a**) Device 1. (**b**) Device 2. (**c**) Device 3.

Moreover, a series of bias-repeatability experiments were conducted on three vacuum-packaged and three non-vacuum-packaged devices to study and investigate further the effect of vacuum packaging and a thermal isolation platform on the MEMS gyroscope’s performance. The results of the three vacuum-packaged devices are shown in Figure 12a–c, which reveals that the target bias repeatability has been achieved with a controlled temperature at 60 °C.

**Figure 13:**
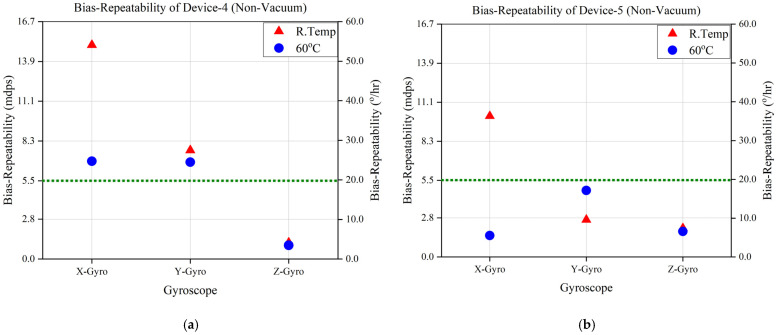

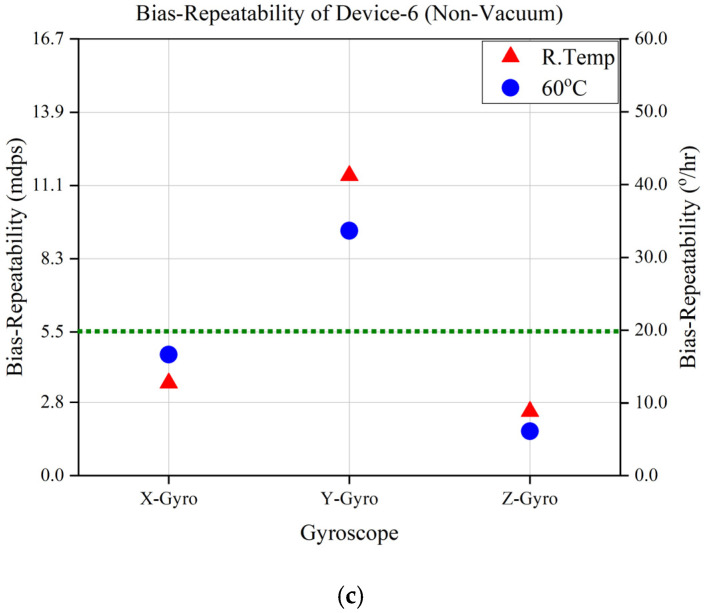
Bias-Repeatability Analysis of Non-Vacuum-Packaged Devices at Room Temperature and 60 °C. (**a**) Device 4. (**b**) Device 5. (**c**) Device 6.

Similarly, the results of non-vacuum-packaged devices are shown in Figure 13a–c, where it can be seen that bias repeatability in some devices was improved with temperature control but was still unsatisfactory compared with vacuum-packaged devices. The temperature variations in both kinds of devices (i.e., vacuum-packaged and non-vacuum-packaged) have been reduced and controlled by the micro-oven, as seen in the bias-repeatability test summary in Table 2. The bias-repeatability and temperature variation results validate the significance of the ovenization and vacuum-packaging technique for the performance improvement of MEMS IMUs.

## 5. Conclusions

This article presents a micro-oven controlled system with vacuum packaging for the commercial MEMS IMUs from BOSCH “BMI 088”. The temperature variations significantly impact the performance of MEMS IMUs, especially gyroscopes, and the bias stability is strongly dependent on temperature variations. The temperature variations have been reduced and compensated using a locally integrated heater in this research work.

It was seen experimentally that the bias repeatability of the MEMS gyroscope is affected by a change in temperature. This was successfully compensated by controlling the temperature with the help of a designed micro-oven. The designed micro-oven has low power consumption, operating at 5 V as provided by the DIO lines of the “NI 8452” device, and the overall design has a small footprint area of $1.5\times 1.5{\;\mathrm{cm}}^{2}$. Using a LabVIEW-based automatic test system, the device was interfaced and tested through an “NI 8452” device.

This work has tested two types of ovenized MEMS IMUs—vacuum-packaged and non-vacuum-packaged. Both kinds of devices were tested at room temperature, as well as at a controlled temperature of 60 °C. Experimentally, it was revealed that the bias repeatability of the devices had been improved. The target specifications were significantly achieved, making the devices suitable for navigation. Furthermore, a comparison of the vacuum-packaged and non-vacuum-packaged devices was performed, showing that the bias repeatability of the vacuum-packaged devices had been improved by more than 60%. It was also noted that temperature compensation is required, and vacuum packaging is mandatory for bias-repeatability improvement.

The temperature variations and bias-repeatability results validated the significance and need for the vacuum-packaging and ovenization method for the performance improvement of MEMS IMUs. Moreover, the results and processing time have proven the significance of the developed automatic test system to increase and improve the mass production of MEMS IMUs.

## Figures and Tables

**Table 1:** Experiment Conditions and PID Parameters.

Parameters	Values	Remarks
Output Data Rate	100 Hz	Correspond to loop time 10 msec, minimum possible value
Duty Cycle Range	0–80%	Test range, where temperature rises to a maximum of 91 °C
P	0.4	Proportional gain
I	0.365	Integral gain
D	0.074	Derivative gain

**Table 2:** Summary of the Bias-Repeatability Analysis.

BMI088		Bias-Repeatability [mdps]			Temperature Variations [°C]	Remarks
Device	Temperature Status	Gyroscopes				
		X	Y	Z		
Device-1	Room Temp	12.868	5.899	3.499	1.598	Vacuum-Packaged
	60 °C	0.610	2.803	1.483	0.002	
Device-2	Room Temp	5.774	5.711	3.468	1.744	
	60 °C	1.283	2.387	2.092	0.001	
Device-3	Room Temp	4.734	9.458	1.817	1.766	
	60 °C	1.515	1.131	1.604	0.001	
Device-4	Room Temp	15.050	7.648	1.150	0.777	Non-Vacuum-Packaged
	60 °C	6.882	6.817	0.966	0.001	
Device-5	Room Temp	10.101	2.672	2.070	0.983	
	60 °C	1.541	4.778	1.834	0.001	
Device-6	Room Temp	3.539	11.479	2.459	1.549	
	60 °C	4.630	9.370	1.699	0.001	

## Data Availability

This research is a part of a classified project.
